# Biodegradable Oxygen‐Generating Microneedle Patches for Regenerative Medicine Applications

**DOI:** 10.1002/anbr.202400093

**Published:** 2024-11-27

**Authors:** Lindsay Barnum, Mohamadmahdi Samandari, Yasir Suhail, Steven Toro, Ashkan Novin, Pejman Ghelich, Jacob Quint, Farnooosh Saeedinejad, Manu Komma, Kshitiz, Ali Tamayol

**Affiliations:** ^1^ Department of Biomedical Engineering University of Connecticut Health Center Farmington CT 06030 USA; ^2^ Department of Biomedical Engineering University of Connecticut Storrs CT 06269 USA

**Keywords:** biomaterials, GelMA, microneedles, oxygen‐generating materials, wound healing

## Abstract

Upon injury, regenerating skin is metabolically active and requires oxygen for physiological processes related to wound healing. Such processes can be halted in hypoxic conditions common in chronic wounds. Microneedle arrays (MNAs) have been demonstrated to improve therapeutic delivery and wound healing. Recently, few studies have explored the use of oxygen‐releasing MNAs; however, they involve complex manufacturing and handling and fail to eliminate cytotoxic byproducts. To address these challenges, biodegradable and mechanically robust gelatin methacryloyl‐based MNAs are developed that can penetrate the tissue and release oxygen upon exposure to interstitial fluid and wound exudates. The oxygen release rate and biocompatibility of the developed MNAs with different compositions are evaluated and optimized. Interestingly, in vitro studies demonstrate that the optimized compositions can release oxygen at therapeutic levels and significantly increase viability of chronically hypoxic cells to match that of normoxic cells. In vivo studies further confirm that the optimized oxygen‐generating MNAs do not cause any harm or impair healing in a murine model of acute skin injury. Additionally, transcriptomic analysis reveals upregulation of key pathways related to fibroblast motility, lipid metabolism, and a marked reduction in inflammatory signaling, all of which contribute to improved wound healing. The developed strategy can introduce new opportunities in elimination of hypoxia and therefore treatment of chronic wounds.

## Graphical abstract

Chronic wounds often suffer from extreme hypoxia, impairing healing. Topically delivered oxygen can fail to diffuse into the wound to reach affected cells. Presented here is a gelatin‐based microneedle array which penetrates tissue and releases oxygen upon contact with liquid. The array rescues survival and gene expression of hypoxic cells.

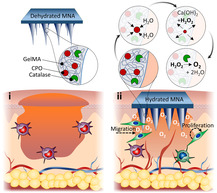

## 1 Introduction

Oxygen serves vital roles in healthy cellular function. Insufficient oxygen affects cellular metabolism, resulting in decreased respiration, and may lead to cell death in highly anoxic conditions.^[^ ^1^ ^]^ Oxygen is particularly important in skin tissue regeneration, where it is required for a number of energy‐intensive and anabolic tasks such as proliferation, epithelialization, inflammation, and collagen deposition.^[^ ^2^ ^]^ Postinjury, the damage to the microvasculature leads to tissue hypoxia which results in stabilization of hypoxia‐inducible factor, which, in turn, promotes angiogenesis, bringing additional oxygenated blood supply to the area.^[^ ^3^ ^]^ In typically healing injuries, hypoxia is mild and transient, but if the tissue is insufficiently vascularized and the hypoxia becomes severe and chronic, tissue regeneration halts and the patient is likely to develop a chronic wound.^[^ ^3^, ^4^ ^]^

Oxygen therapies are widely used and, in some circumstances, have shown clinically relevant improvement of wound healing. Existing oxygen treatments for skin injuries have included hyperbaric oxygen therapy^[^ ^5^ ^]^ and topical oxygen therapy.^[^ ^6^ ^]^ Hyperbaric oxygen therapy involves placing a patient into a chamber filled with pure oxygen at a pressure higher than the atmospheric level and has shown mixed results in increasing wound closure rate depending on the time period considered and etiology of the wound.^[^ ^7^ ^]^ It further suffers from challenges such as high cost and complexity, risk of systemic oxygen toxicity, and lack of delivery specificity.^[^ ^8^ ^]^ While topical oxygen therapy has shown promise in randomized controlled clinical trials^[^ ^9^ ^]^ and overcomes some of the challenges of hyperbaric medicine, it can only penetrate superficial tissue,^[^ ^6^ ^]^ which leaves the majority of the wound bed out of range. This is particularly problematic in chronic wounds with excess necrotic tissue or biofilm burden that is difficult or dangerous to debride. Additionally, while smaller and more portable topical oxygen therapy devices have been developed,^[^ ^10^ ^]^ they still require complex electricity and oxygen supply systems, which can make their deployment in resource‐poor regions difficult. Furthermore, the large footprint of such systems limits their application as wearable therapeutics, restricting patients from their routines. To address these challenges, some groups have developed wearable scaffolds and dressings containing oxygen‐releasing materials to produce oxygen in situ.^[^ ^11^ ^]^

Different scaffolding biomaterials have been used for tissue oxygenation such as oxygen microbubble‐embedded biomaterials,^[11a]^ microalgae patches,^[11b]^ and hydrogel microcarriers with triggered hemoglobin‐bound oxygen release.^[11c]^ Another strategy is to use chemical reagents that decompose into oxygen. Hydrogen peroxide (H_2_O_2_) solution has been used previously to release oxygen topically onto the wound bed. However, its oxidative stress requires mitigation techniques to prevent burst release, such as complex and multistep microsphere encapsulation^[11d]^ or microfluidic pumping, which presented leakage issues that at times negatively impacted wound healing.^[11e]^ The difficulties of dealing with H_2_O_2_ solution make solid chemical precursors more appealing. Calcium peroxide (CPO) is one of the most widely used oxygenating chemicals, which can generate H_2_O_2_ and oxygen upon exposure to aqueous environments, with further oxygen production through H_2_O_2_ decomposition requiring a catalyst.^[^ ^12^ ^]^

While the aforementioned treatments can offer wearable platforms for topical tissue oxygenation, they still suffer from poor tissue penetration. Particularly, chronic wounds present significant challenges in delivering drugs to the intended cells due to exudation and biofilm presence.^[^ ^13^ ^]^ To bypass these barriers, microneedle arrays (MNAs) were selected as the platform to deliver oxygen into the wound bed. MNAs are a growing area of interest for the treatment of wounds,^[^ ^14^ ^]^ and we have previously shown that using MNAs to increase the depth of delivery improves the distribution of therapeutics in a wound and enhances the speed and quality of healing, while using injection creates poorer lateral distribution, delivery of drugs beyond the injured area, and worse healing outcomes.^[^ ^15^ ^]^ In the care of many chronic wounds, nonviable tissue is removed before other treatments are applied. However, in wounds suffering from local ischemia, debridement cannot be performed until local blood flow is restored.^[^ ^16^ ^]^ This means that wounds with the greatest need for oxygen supply often have the additional obstacle of necrotic or sloughy tissue, making MNAs that can bypass those barriers an attractive choice for oxygen delivery.

MNAs fabricated from a broad range of materials and incorporating a variety of therapeutics have been proposed to help treat chronic wounds, from antibacterial compounds^[^ ^17^ ^]^ to hormones^[^ ^18^ ^]^ to stem cell‐derived exosomes,^[^ ^19^ ^]^ with promising new manufacturing techniques being developed.^[^ ^20^ ^]^ While antioxidant MNAs have been presented,^[^ ^21^ ^]^ only a few MNA‐based oxygen delivery platforms have been developed.^[^ ^22^ ^]^ These works have suffered from complicated fabrication and storage methods and insufficient clearance of intermediate reactive oxygen species.

Based on the limitations of the previous studies, we sought to create an oxygen‐releasing MNA that was simple, fast, and low‐cost to fabricate, store, and use. In addition, we wanted to increase the release duration seen in previous CPO‐based work and ensure that cytotoxic H_2_O_2_ was decomposed immediately. Catalase is a very effective enzyme for the decomposition of H_2_O_2_^[^ ^23^ ^]^ and has been used in a number of oxygen‐releasing biomaterials,^[^ ^24^ ^]^ and was selected for this work to manage H_2_O_2_ concentration. In this study, we engineered biodegradable MNAs that generate sustained oxygen release without maintaining a significant amount of H_2_O_2_. The needles were fabricated by first incorporating CPO and catalase into a solution of gelatin methacryloyl (GelMA), a frequently used, photocrosslinkable biomaterial with high strength and stiffness when dry and soft, compliant properties when hydrated.^[^ ^25^ ^]^ The CPO and catalase‐loaded GelMA prepolymer solution could then be easily formed into desired shapes through molding prior to crosslinking, providing a quick and easy fabrication process. While degradation testing showed the material will degrade over time, the produced MNAs did not degrade rapidly in vivo and would therefore be removable during dressing changes if needed. We measured the rate of oxygen generation and investigated the benefit of oxygen‐eluting MNAs in rescuing hypoxic cells in vitro. We also confirmed the biocompatibility of the engineered MNAs in a murine model with an acute, full‐thickness skin wound. RNA sequencing showed that the developed treatment could activate pathways associated with restoring lipid metabolism, increasing cellular migration, and decreasing inflammatory cell migration. Through a simple micromolding method, shelf‐stable, hydrogel‐based oxygen‐eluting MNAs were developed to bypass local barriers present in chronic wounds and deliver oxygen to severely hypoxic cells.

## 2 Results and Discussion

**Figure 1:**
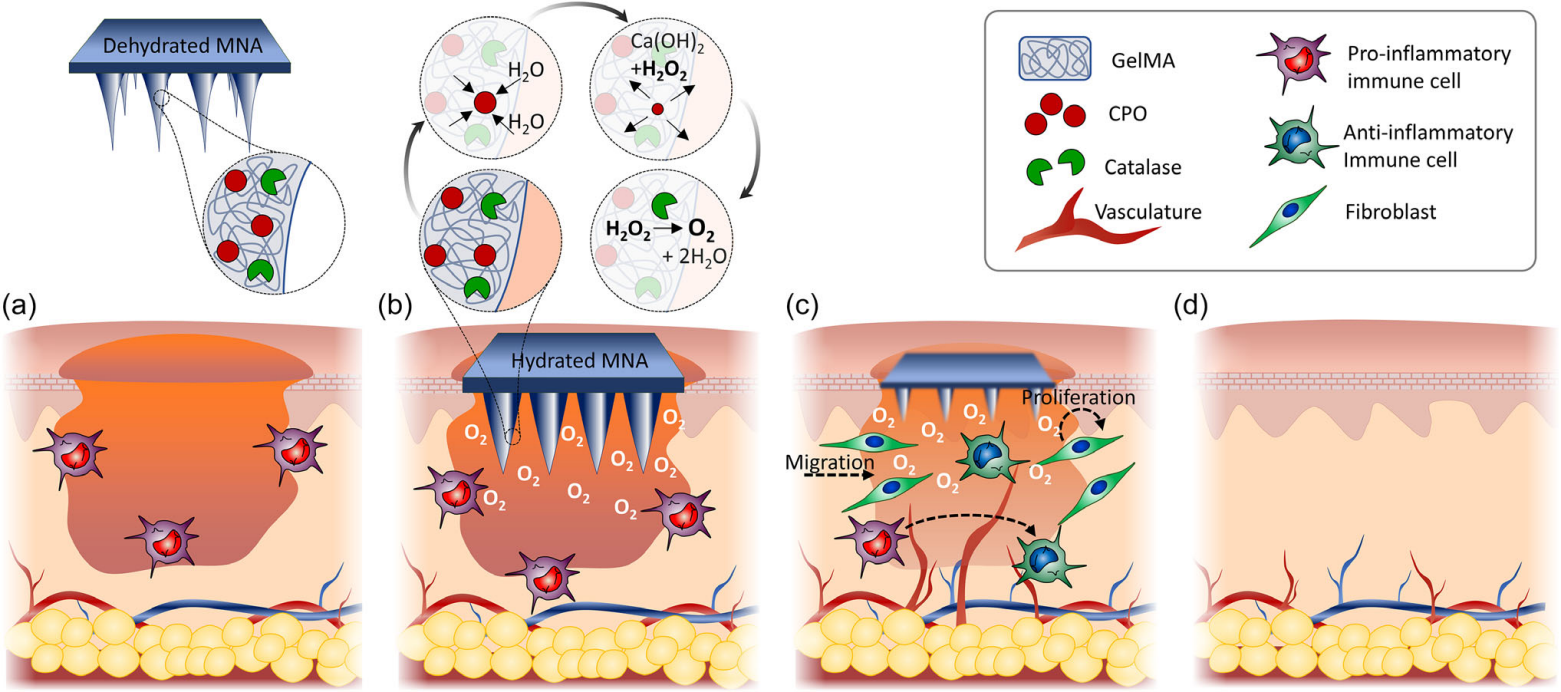
Oxygen‐generating MNAs for wound healing applications. a) Dried GelMA MNAs containing CPO and catalase were used in this study. b) Upon exposure to the aqueous environment in the tissue, the dried MNA hydrates and exposes embedded CPO to water. This results in the reaction of CPO with water, generating H_2_O_2_. The H_2_O_2_ will then rapidly decompose into water and oxygen, as a result of catalase action. c) The released oxygen in the hypoxic wound bed can enhance cell migration and proliferation, as well as inflammation resolution, toward d) the healing of the wound. This figure is reproduced in an edited format with permission from John Wiley and Sons.^[^ ^36^ ^]^

**Figure** **1** illustrates the concept of the work and the MNA mechanism of action in the treatment of wounds. In order to produce oxygen, GelMA MNAs are fabricated containing CPO and catalase. Upon contact with wet tissue, CPO reacts with water and produces oxygen and H_2_O_2_. The catalase works to rapidly decompose the H_2_O_2_ into water and additional oxygen. The oxygen produced in these reactions is released into the wound environment to minimize local hypoxia and support cellular recovery.

**Figure 2:**
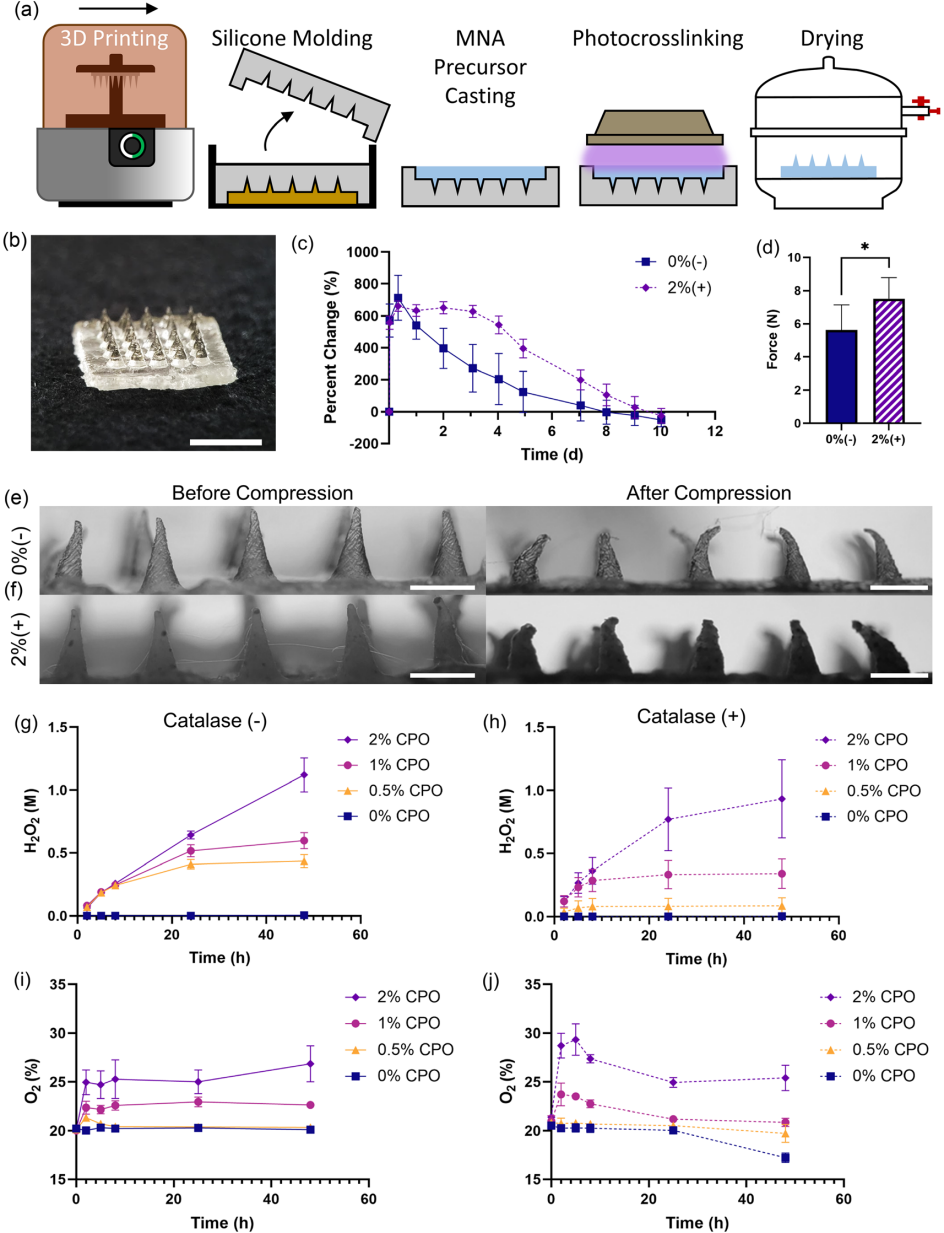
MNA fabrication and physical characterization. a) Illustration of the MNA fabrication process: the designed MNA is 3D printed and used as a master mold. Then, a negative mold is formed through silicone rubber casting and filled with hydrogel prepolymer. Air bubbles are removed and the prepolymer is photocrosslinked. The sample is then unmolded and dried in a desiccator to produce the final MNA. b) A hydrogel MNA before dehydration. Scale bar represents 5 mm. c) Graph plotting percentage mass change from initial dry mass of 0%(–) and 2%(+) material samples undergoing degradation testing in collagenase solution shows successful degradation of both groups. d) Graph comparing maximum compressive force generated during 30% compression of dehydrated MNAs, with the 2%(+) array demonstrating greater stiffness. e,f) Representative images of 0%(–) and 2%(+) MNAs, respectively, before and after compression. The needles bent without breaking. Scale bars represent 1 mm. Cumulative H_2_O_2_ release from samples at various CPO concentrations g) without catalase and h) with catalase. Real‐time oxygen concentration plotted over time from various CPO concentrations i) without catalase and j) with catalase. Increasing CPO concentration increased the rate of H_2_O_2_ and O_2_ production, while the inclusion of catalase decreased H_2_O_2_ release and increased O_2_ production. A summary of *P*‐values for hydrogen peroxide release is presented in Table S1, Supporting Information and those for oxygen release in Table S2, Supporting Information.

To fabricate MNAs, concentrated solutions of CPO and catalase were added to the GelMA prepolymer before crosslinking. Eight formulations of MNA precursor, introduced in **Table** **1**, were tested in this study to optimize oxygen production and biocompatibility. The concentration of catalase was maximized to decompose the greatest amount of H_2_O_2_ possible. Above the values of CPO and catalase used, the MNAs became increasingly sticky at the molding interface and could not be removed cleanly, likely due to poor crosslinking through the opaque solutions. For this reason, no higher concentrations of catalase could be incorporated to create formulations with more CPO and subsequently greater oxygen release. To create the GelMA MNAs, we utilized a simple molding fabrication method (**Figure** **2**a). Positive master molds were designed in SolidWorks, 3D printed, and then cast in silicone rubber to create flexible negative molds. GelMA prepolymer solution, at a final concentration of 15% w/v, was then pipetted into the mold and debubbled. The solution was then crosslinked through blue light exposure to fabricate the hydrogel MNAs (Figure 2b). The MNAs were then dried to produce final samples for future assessments. A peroxide assay was performed on H_2_O_2_ solution treated with desiccated and freshly prepared material samples to ensure that the drying process did not negatively impact catalase activity, with results showing complete elimination of H_2_O_2_ by both groups at the 24 h endpoint (Figure S1, Supporting Information). A 0%(–) negative control even showed a slight decrease in peroxide concentration just from exposure to GelMA. The MNAs shrink in volume by approximately half from their hydrated state, decreasing the final length of the needles and increasing their sharpness. The postdrying length of an individual microneedle was an average of 747 μm (Figure S2, Supporting Information). The MNAs swell rapidly when exposed to water to create a compliant tissue interface (Figure S3, Supporting Information). Although the material swells about 6 times from its original weight, the MNA is not expected to strain the surrounding tissue, due to the topical application method; however, detachable or rapidly degradable backings could be incorporated if this became a challenge. While catalase forms a solution in phosphate‐buffered saline (PBS), CPO remains a suspension and dissolves slowly over time. To ensure that CPO was well distributed in the samples, phase micrographs were taken of the backing (Figure S4a, Supporting Information) and a cross section of the backing and a needle (Figure S4b, Supporting Information), showing homogenous distribution of CPO particles. A biomimetic degradation assay was performed to evaluate the biodegradability of the MNA through submersion in a collagenase solution. The arrays degrade over time, and while there was a delay seen in the 2%(+) group, the group with the highest concentration of additives, compared to the 0%(–) group, the difference was not statistically significant, and by the end of the experiment, there was no noticeable difference in total degradation (Figure 2c). Scanning electron microscopy (SEM) was performed to determine if the incorporation of CPO and catalase would have an impact on needle structure. The 0%(–) needles (Figure S5a, Supporting Information) showed no notable difference from the 2%(+) group (Figure S5b, Supporting Information). Imaging of the two groups showed similar shape fidelity. To ensure that the mechanical properties of the MNAs were similarly unaffected by incorporation of CPO and catalase, compression testing was performed comparing 0%(–) MNAs and 2%(+) MNAs. The MNAs were compressed to ≈30% strain, and the maximum compressive force was recorded (Figure 2d). The addition of the CPO and catalase resulted in a slight increase in maximum force, which could make skin penetration easier. Both groups demonstrate maximum force values greater than the force required to penetrate skin.^[^ ^26^ ^]^ Images captured after compression show that the increase in stiffness did not result in brittleness (Figure 2e,f) that might have made the MNAs harder to handle or apply. Both groups exhibited bent rather than crushed or broken off tips after compression. Finally, ex vivo pig skin penetration testing was performed with 0%(–) MNAs. Tissue sections were H&E stained to demonstrate that the needles could pierce the epidermis (Figure S6, Supporting Information).

**Table 1:** Formulation group names.

	Without 1% catalase	With 1% catalase
0% CPO	0%(–)	0%(+)
0.5% CPO	0.5%(–)	0.5%(+)
1% CPO	1%(–)	1%(+)
2% CPO	2%(–)	2%(+)

H_2_O_2_ and oxygen release studies were conducted to assess the impact of CPO and catalase on the production of H_2_O_2_ and oxygen. To track H_2_O_2_ release, samples with different compositions were placed in PBS and monitored over time. At each time point, the PBS was sampled and refreshed completely. The collected PBS samples were then analyzed using a colorimetric peroxide detection kit. As expected, H_2_O_2_ production increased with increasing CPO concentration in the MNAs, both without the presence of catalase (Figure 2h) and with catalase (Figure 2i). However, at all CPO concentrations, the inclusion of catalase decreased the amount of H_2_O_2_ measured. The H_2_O_2_ in solution at each time point (Figure S7a,b, Supporting Information) shows that while the groups without catalase were still producing additional H_2_O_2_ at 24 h, in the groups with catalase, H_2_O_2_ decomposition overcame production within the first day. These data also show that at 2% CPO, the amount of catalase present in each sample is no longer sufficient to break down all of the H_2_O_2_ being released. Even with catalase, the 2% CPO samples were generating H_2_O_2_ after 48 h. Greater values of catalase may have been able to decompose the additional H_2_O_2_; however, higher concentrations of CPO and catalase interfered with blue light penetration, resulting in poorly crosslinked MNAs that stuck to the molds. *P*‐values for H_2_O_2_ release comparisons are presented in Table S1, Supporting Information.

To measure oxygen production, MNAs were fabricated and then sealed in glass bottles containing optical oxygen sensor dots for the full study period. A sensor probe was used to read the oxygen level through the sensor dot at each time point without opening the bottles. Real‐time oxygen monitoring showed delineation between different concentrations of CPO, with differences apparent on the first day (Figure 2j,k). The majority of oxygen release took place within the first 8 h, after which the concentration was mostly stabilized. In the groups with catalase, there was a larger increase in oxygen concentration through the initial time points, followed by a drop to the final concentration reached by the 24 h time point. The initial increases in percent oxygen for the 1% and 2% groups represent an increase of ≈22 mmHg and 65 mmHg, respectively, enough to move tissue from the ischemic range, 5–30 mmHg, and into the healthy tissue range, 30–60 mmHg.^[^ ^27^ ^]^ This release data has also been presented in Figure S8, Supporting Information with a graph for each CPO concentration, to better visualize the effect of catalase within a CPO group. *P*‐values for O_2_ release comparisons are presented in Table S2, Supporting Information. To account for the drop in oxygen after the initial increase, we explored whether the presence of catalase had an effect on the optical oxygen sensor. As can be seen in Figure S9, Supporting Information catalase in the solution causes a decrease in the measured oxygen level after 24 h compared to a plain PBS control. There is a greater effect when the catalase is mixed directly into the PBS, but still a noticeable decrease when it is released from a GelMA sample. This effect is delayed ≈24 h, and the cause has not yet been determined. Future studies will identify alternate oxygen measurement techniques to elucidate this phenomenon. It should be noted that the measurements were conducted from submerged MNAs and in real applications where the constructs are interfaced with small amounts of interstitial fluids, the release could be more gradual and concentrated.

Overall, increasing the concentration of CPO increases both H_2_O_2_ and oxygen production from the GelMA samples, with the inclusion of catalase decreasing H_2_O_2_ in solution and increasing oxygen. By manipulating the concentration of CPO and catalase, H_2_O_2_ presence can be limited, and oxygen release can be controlled to maximize the benefit to the cells.

**Figure 3:**
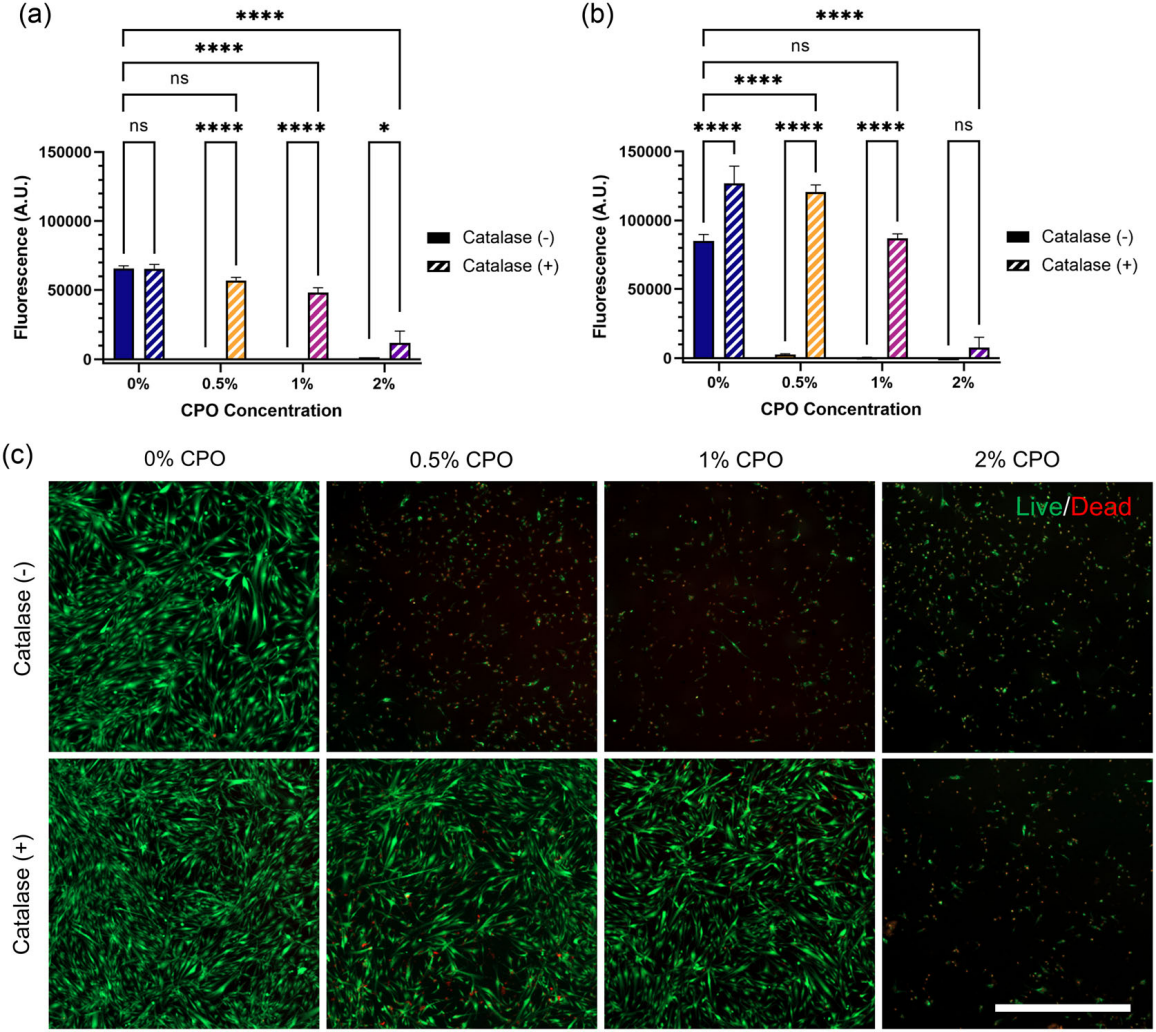
Cytocompatibility characterization. a,b) PrestoBlue viability assay results following direct exposure of HDFs to samples on a) day 1 and b) day 3 show that catalase is necessary for cell survival and that 1% CPO is the greatest concentration cells can survive. A full factorial comparison was completed, but only comparisons within CPO concentrations and to the negative control are shown. c) Representative day 1 Live/Dead images of HDFs, where live cells appear in green and dead cells in red. Scale bar represents 500 μm. The Live/Dead results for day 3 are provided in Figure S10, Supporting Information.

All combinations of CPO and catalase were evaluated for their effect on cells in a normoxic environment to determine the limit of H_2_O_2_ release that would be harmful to cells (**Figure** **3**). PrestoBlue (Figure 3a,b) and Live/Dead (Figure 3c and Figure S10, Supporting Information) assays were conducted 1 and 3 days after the incubation of the samples directly inside the culture media of human dermal fibroblasts (HDFs). Background testing verified that the samples did not interfere with PrestoBlue signal readings (Figure S11, Supporting Information). Both assays show that without catalase, no amount of CPO is survivable. Quantitative results, on both day 1 and day 3, demonstrated that the cells exposed to 0.5, 1, and 2% CPO, in the absence of catalase, had negligible viability, while imaging results showed only a few live cells and poor morphology in these groups. Also, at both time points, 2% CPO was harmful to the cells even with the inclusion of 1% catalase. In 2% CPO groups, the PrestoBlue signal started low and decreased over time, showing no growth over the 3 day experimental period. This result was expected based on the H_2_O_2_ release experiments, demonstrating the insufficiency of the catalase concentration to decompose H_2_O_2_ released from 2% CPO. For 0.5% and 1% CPO, however, the catalase effectively broke down the released H_2_O_2_ and protected the cells. On day 1, the 0.5%(+) group was not statistically distinct from the 0%(–) group, but by day 3, the 0.5%(+) group showed significantly higher viability. The 1%(+) group showed similar growth, starting with lower viability than the control and then, by day 3, showing no statistical difference. Interestingly, while the 0%(+) group is indistinguishable from 0%(–) on day 1, the signal from the cells was significantly higher by day 3, suggesting that catalase alone may have some positive effect on the cells. The effect of catalase as an enzyme that decomposes H_2_O_2_ is well established^[^ ^28^ ^]^ and its use as a therapeutic to treat wounds has been explored in a number of studies.^[^ ^29^ ^]^ Recent works have presented a number of “catalase‐like” constructs incorporated into wound dressings that relieve oxidative stress and improve wound healing.^[^ ^30^ ^]^ While we are not targeting catalase as our primary therapeutic in the MNAs, these experiments showed that catalase is an essential component when developing CPO‐laden, oxygen‐releasing materials. Without catalase, the H_2_O_2_ that is initially produced rapidly kills cells. While this may provide some antibacterial effect in vivo, the harm to cells that are already under oxidative stress would not improve the wound healing. Regardless of whether H_2_O_2_ is administrated directly or formed in an intermediate step, if it is used in oxygen‐producing dressings, it should be catalyzed before it can come into contact with the wounded tissue. Previous works have failed to address this critical need for an H_2_O_2_ catalyst in CPO‐based oxygen MNAs.^[22c]^

**Figure 4:**
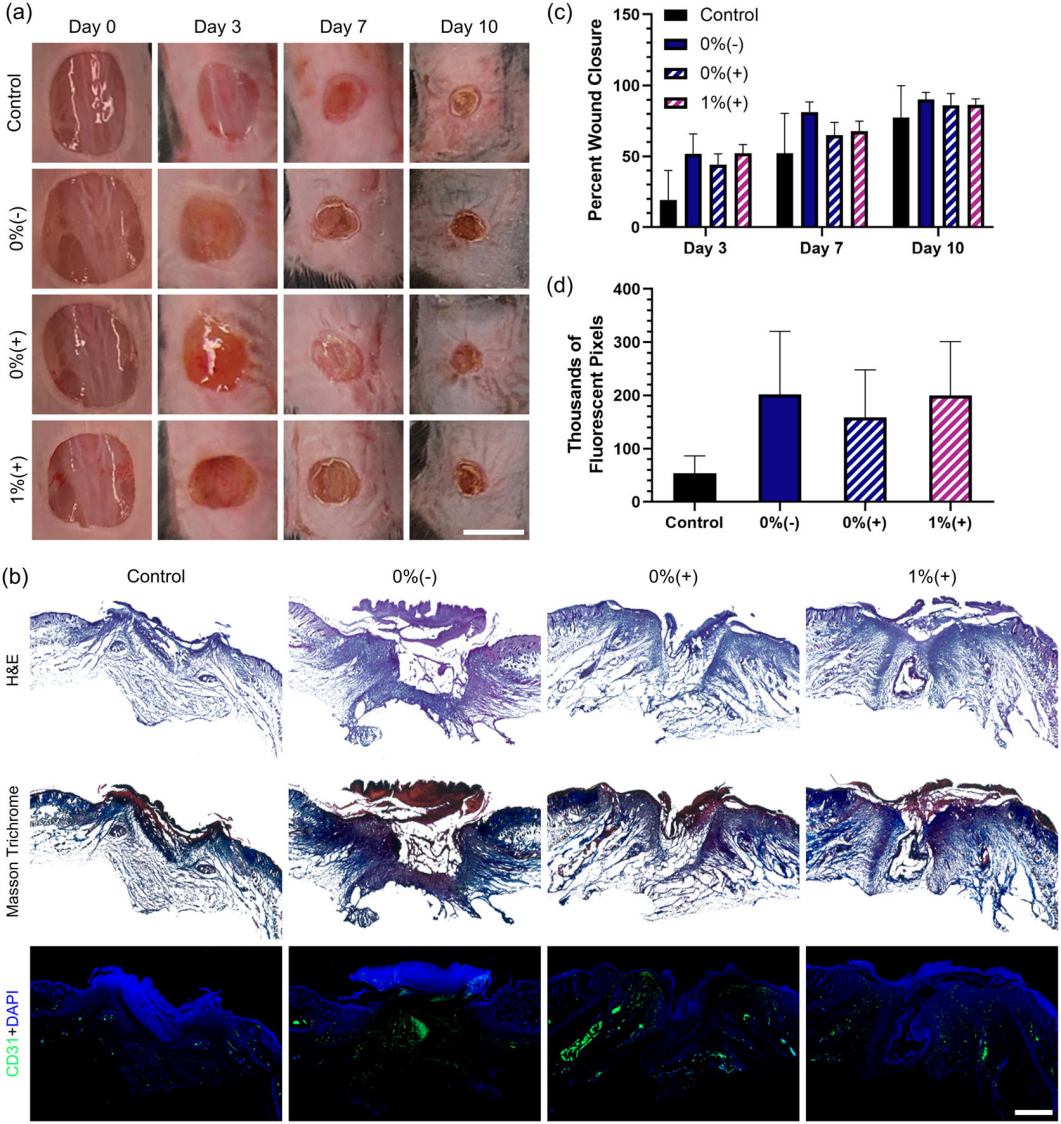
Assessment of in vivo biocompatibility of the oxygen‐eluting MNAs. a) Representative macroscopic wound healing images from each treatment group over the 10 day study period show that wound closure was not impaired by the application of the MNAs. Scale bar represents 1 cm. In this study, Tegaderm was used as a commercial control, representing a standard‐of‐care treatment. b) Representative H&E, Masson Trichrome, and CD31 + DAPI sections of wound tissue harvested on day 10 show similar morphology between groups. Scale bar represents 1 mm. c) Percent wound closure relative to initial day 0 wound size. d) The number of CD31 positive pixels in the wound area of tissue sections indicates slightly stronger angiogenesis in the treatment groups.

Some previous studies that have used H_2_O_2_, or oxygen‐generating materials that produce H_2_O_2_, showed some level of toxicity in vitro or in vivo.^[11e]^ While the inclusion of catalase in our optimized dressing was shown to eliminate the negative effects of reactive oxygen species in vitro, we wanted to verify the safety of the dressing in vivo. To achieve this, an animal study was conducted using healthy wild‐type mice given full thickness dorsal skin wounds. Animals were treated with 0%(–), 0%(+), and 1%(+) MNAs (Figure S12, Supporting Information), or with only a Tegaderm wrap, acting as a standard‐of‐care control. All groups demonstrated a similar healing rate compared to those in the control group, with a small trend showing slightly faster healing in the treatment groups (**Figure** **4**a,c). While this model is not appropriate to show efficacy of the oxygen treatment, it is clear that the developed dressing is not inhibiting wound closure. H&E and Masson Trichome staining of tissue sections harvested on day 10 of the study showed comparable morphology between the four groups, without notable differences in collagen deposition or epidermal thickness on the wound edges (Figure 4b). CD31 staining was also performed to examine angiogenesis, and although there was a trend indicating higher expression in the treatment groups over the control, there was not a statically significant difference (Figure 4b,d). In wound healing, some hypoxia is necessary to facilitate blood vessel growth, but our dressing avoid any inhibitory effect. In this study, we used a healthy wound healing model to confirm that our dressings were safe for in vivo application. In future work, the efficacy of the system should be tested in animal wound models with chronic hypoxia, like in diabetic chronic wound models, or in a peripheral ischemic model, where cells have been impaired by lack of oxygen. While our studies on healthy mice confirm the biocompatibility of the strategy, wounds with pathologically chronic hypoxia may show improved healing with delivery of oxygen.

Anoxia, or near‐complete scarcity of oxygen, can result in shutdown of cellular respiration, and most anabolic reactions necessary for cell growth and proliferation. Therefore, we sought to assess if the administration of CPO on hypoxic cells could rescue the changes in cellular metabolism and metabolic substrate utilization. The previous study indicated that with catalase, 1% CPO is the highest survivable concentration of CPO in a normoxic environment. While the performance of 0.5% GelMA with catalase was higher, this was in a normoxic environment. In chronic hypoxia, the goal is to generate as much oxygen as possible. The 0.5%(+) group only generated sufficient oxygen to cause an increase of about 2 mmHg, whereas the 1%(+) group creates an increase of about 22 mmHg, a sufficient value to bring tissue oxygen levels from an ischemic to a healthy state.^[^ ^27^ ^]^ Therefore, 1% CPO was selected for hypoxia rescue experiments.

**Figure 5:**
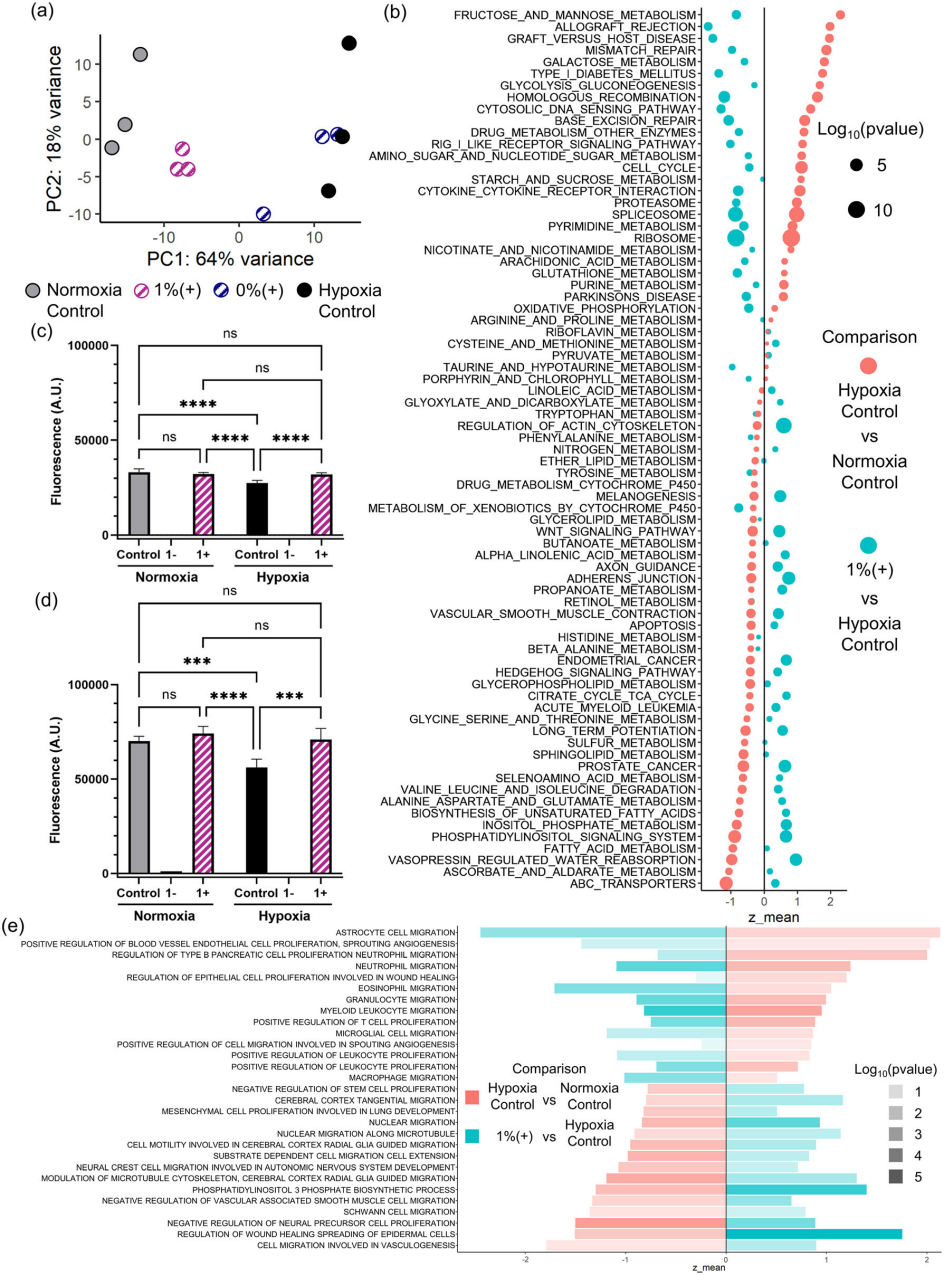
In vitro hypoxia rescue. a) Principal components for the 500 most variant genes in the RNAseq of the samples show the overall partial reversal of the effect of hypoxia. b) Metabolic and other selected gene ontology sets and the mean regulation for the two comparisons also show overall reversal of the effect of the MNA treatment. c) Day 1 and d) day 2 PrestoBlue viability assay results comparing normoxic and hypoxic culture conditions during material exposure show the decrease in activity brought on by chronic hypoxia and the rescue effect of the MNAs. e) Migration and other selected gene ontology sets and the mean regulation comparing hypoxia control to normoxia control and the 1%(+)‐treated group in hypoxia to hypoxia control show increased expression of fibroblast migration pathways and decreased expression of inflammatory signaling pathways.

To measure the systemic effect of CPO on gene expression in cells subjected to hypoxia, we maintained HDFs in 5% O_2_ for 2 days, without treatment (hypoxia control) or treated with 1%(+) constructs. 0%(+)‐treated cells in hypoxia and untreated cells in normoxia were also used as controls. The RNA from these groups was isolated and then sequenced. Principal component analysis (PCA) of individual samples showed large transcriptomic variance between normoxia and hypoxia, as expected, with the 1%(+) group showing a large reversal of transcriptional signal in hypoxic cells toward the direction of normoxia (**Figure** **5**a). As PCA showed that the combination of CPO and catalase reversed most of the hypoxia generated changes in gene expression, we investigated these reversals in greater detail. We identified the gene‐sets that had increased (or decreased) in hypoxia versus normoxia but showed a significant reversal in the presence of our 1%(+) samples (Figure 5b). Gene‐sets associated with hypoxia mediated metabolic effects were reversed by the 1%(+) samples. These included the large, expected increase in glycolysis by hypoxia, as well as nucleotide metabolism. Gene‐sets for galactose metabolism, glycolysis, gluconeogenesis, as well as starch metabolism were increased in hypoxia, but reversed by the CPO and catalase treatment. In contrast, we found that gene‐sets associated with the metabolism of lipids and various amino acids had decreased in hypoxia but were restored by our treatment. These included gene‐sets associated with the metabolism of signaling sphingolipids, fatty acids, glycerophospholipids, as well as anabolic reactions of fatty acids. Metabolic activity of nearly all amino acids which had decreased in hypoxia was restored by CPO. What was remarkable was the marked reduction in lipid‐mediated signaling in hypoxia, rescued by CPO. These included the inositol phosphate and phosphatidylinositol signaling, also supported by the gene‐sets related to cell migration, which showed a similar trend. Additionally, gene‐set enrichment analysis showed strong upregulation of the KEGG pathway for actin cytoskeleton regulation, likely indicating increased contractile activity (Figure S13, Supporting Information). Cell proliferation‐ and cell cycle‐related pathways were reversed by the addition of the MNAs compared to hypoxia (Figure S14, Supporting Information). As transcriptomic data strongly showed that proliferation was increasing in fibroblasts in response to hypoxia, and partially reversed by our treatment, we confirmed the metabolic changes accompanied by these stimulations. It should be noted that fibroblast activity cannot not be viewed as an unmitigated good in the area of wound healing because it can lead to keloids and excessive scarring if not maintained in balance. Therefore, the reversal of this process by oxygen‐eluting MNAs can potentially reduce scarring and keloid formation. It is noteworthy that in our pilot mouse study, neither keloid development nor unusual levels of scarring were observed.

We then sought to determine how these differences in gene expression would play out in overall cell behavior. As previously discussed, some level of hypoxia can be stimulating to cells, but when it goes on for too long, it can inhibit growth.^[^ ^31^ ^]^ The RNA analysis results suggested that after a certain point, hypoxia harmed the metabolism and function of the fibroblasts. In this study, parallel experiments were carried out in normoxia and chronic hypoxia over 2 days (Figure 5c,d), with exposure to hypoxia beginning sooner than in the RNA experiment. 1%(+)‐ and 1%(–)‐treated cells were compared to an untreated cell control. On both days and in both normoxia and hypoxia, 1%(–) samples killed all the cells. The greater need for oxygen in hypoxic culture did not outweigh the cellular damage of H_2_O_2_ produced by the samples. In the negative control groups, there was significantly lower metabolic activity in hypoxia compared to normoxia. This was apparent by day 1 and persisted on day 2. In the normoxia group, there was no significant difference between the negative control and the 1%(+) treatment group on either day, showing that providing additional oxygen to cells without a deficit does not increase their activity. In the hypoxia group, however, the 1%(+) treatment group did not show the same decrease in metabolic activity exhibited by the negative control. It performed significantly better, with a signal equivalent to that of both groups in normoxia. The MNAs are also expected to release some Ca^2+^ ions, which have the potential to increase fibroblast activity; however, given that there is an increase in signal after treatment only under hypoxic conditions, oxygen is thought to be the primary therapeutic agent in this system. A similar experiment was performed with human umbilical vein endothelial cells, which showed a lower viability with exposure to hypoxia (Figure S15a, Supporting Information) and higher viability in hypoxia when exposed to 1%(+) treatment (Figure S15b, Supporting Information), demonstrating the cells responsible for angiogenesis also respond positively to the MNAs. These results demonstrate the deleterious effect of chronic hypoxia on cellular metabolic activity and the ability of our system to provide a physiologically relevant concentration of oxygen that can rescue cells under chronic hypoxia, as is common in chronic wounds. In combination with the RNA results, we can see that while hypoxia can increase fibroblast proliferation, over time, it can suppress metabolic activity.

Gene expression analysis suggested that CPO and catalase could significantly reverse the hypoxia‐induced reduction in cell migration, as well as cell signaling associated with physiological wound healing. Both Wnt and hedgehog signaling are crucial for normal, nonregenerative wound repair,^[^ ^32^ ^]^ and both pathways were upregulated by 1%(+) treatment (Figure 5e). Wound healing and closure require fibroblasts to migrate to the site of the wound, activate and proliferate, and then lay down the matrix for wound closure. While acute hypoxia is a crucial determinant of the inception of wound healing response, chronic hypoxia or anoxia can inhibit this process, resulting in suboptimal fibroblast proliferation, migration to the site, and incomplete wound closure. Gene ontology analysis of dermal fibroblasts subjected to 5% O_2_ in vitro and then to 1%(+) samples showed a range of responses. Pathways associated with cellular migration and wound healing response were upregulated by our treatment. Additionally, many pathways associated with signaling for the migration of inflammatory cells were upregulated by hypoxia but were reversed by CPO and catalase. This is a promising combination for improving healing in chronic wounds, where there is a lack of fibroblast activation and an overactive inflammatory response. However, the assessment of inflammation level changes requires more in‐depth analysis involving immune cells such as monocytes and macrophages that are important players in wound healing.

## 3 Conclusion

In this work, we present the rapid and facile fabrication of GelMA MNAs, loaded with CPO and catalase, to controllably release oxygen without maintaining harmful levels of H_2_O_2_. Release profiles for H_2_O_2_ and oxygen were examined, and the effect of different concentrations of CPO and catalase was established. HDFs were exposed to the various MNA formulations, and 1%(+) was selected as the composition that produced the most significant concentration of oxygen without harmful levels of H_2_O_2_. Further, the safety of the dressing for use in vivo was shown in an acute murine wound healing model. The MNAs were then tested for their ability to rescue cells negatively impacted by chronic hypoxia exposure. Gene expression indicated a large transcriptomic variance between cells cultured in normoxic and hypoxic conditions, with 1%(+) MNA treatment of hypoxic cells creating a strong reversal toward normoxia. Key gene‐sets that were upregulated, such as those related to glycolysis, or downregulated, like those associated with lipid metabolism, by hypoxia showed strong recovery in the 1%(+)‐treated samples. Additionally, in hypoxia, gene ontology analysis showed downregulation of fibroblast migration pathways and upregulation of inflammatory signaling pathways, which were reversed in the treatment group. This platform has the potential to be customized in size, configuration, and oxygen content to meet patient specific needs. Future work would explore different materials and crosslinking methods, expand on the demonstrated cell rescue effect to explore interplay with immune cells, examine synergistic effects between oxygen‐eluting and proangiogenic therapeutics, and utilize a more representative animal model, such as a diabetic or ischemic wound, to show the efficacy of the dressing in vivo.

## 4 Experimental Section

4.1

4.1.1

##### Materials

Gelatin, methacrylic anhydride, lithium phenyl‐2,4,6‐trimethylbenzoylphosphinate (LAP), CPO, and catalase were purchased from Sigma–Aldrich. Basal fibroblast media and low serum bullet kit were purchased from American Type Culture Collection. The Live/Dead Viability/Cytotoxicity Kit, Pierce Quantitative Peroxide Assay Kit, PrestoBlue HS Cell Viability Reagent, and Goat anti‐Rabbit IgG (H + L) Highly Cross‐Adsorbed Secondary Antibody, and Alexa Fluor Plus 488 were purchased from ThermoFisher Scientific. Hematoxylin and Eosin Stain Kit was purchased from Vector Labs. Trichrome, Gomori One‐Step, Aniline Blue Stain Kit was purchased from Newcomer Supply. Primary CD31 antibody was purchased from Lifespan Biosciences.

##### MNA Fabrication

Positive models were designed in SolidWorks and 3D printed on a Formlabs Form3B SLA printer. Negative molds were then cast using a two‐part silicone rubber kit. GelMA was synthesized with a medium degree of functionalization. Briefly, a 10% gelatin solution was prepared and heated until fully dissolved. Methacrylic anhydride, at a rate of 0.125 g per 1 g of gelatin, was added dropwise to the gelatin solution under constant stirring. This was allowed to react for 1 h at 50 °C and was then diluted with prewarmed PBS to halt the reaction. The GelMA solution was dialyzed for 1 week with twice daily water changes to remove any unreacted methacrylic anhydride. After dialysis, the solution was vacuum filtered and aliquoted into centrifuge tubes and then freeze‐dried (FreeZone 2.5 L − 50 °C Benchtop, Labconco) for 1 week. Tubes were stored in −20 °C until use. GelMA solution was prepared in PBS such that after the incorporation of CPO and catalase it would have a final concentration of 15% w/v with 0.067% w/v LAP. CPO and catalase were incorporated from freshly prepared high concentration stock solutions and mixed with the GelMA solution to create the experimental solutions: 0, 0.5, 1, and 2% CPO with and without 1% catalase for a total of eight groups. These solutions were then pipetted into the prewarmed negative molds and a pipette tip was used to remove bubbles from the needle tips. Samples were crosslinked for 3 min with a blue light lamp before use. Drying was accomplished by placing samples in a desiccator overnight.

##### Catalase Activity

30 μL material samples were prepared. There were five groups with four replicates each: dry 0%(–) and 0%(+), fresh 0%(–) and 0%(+), and a plain H_2_O_2_ group that served as a negative control. Dry samples were made the day before the experiment began and desiccated overnight, and fresh samples were prepared immediately preceding the beginning of the experiment. All the samples were added to a 48‐well plate, and then 0.5 mL of 5 mM H_2_O_2_ solution was pipetted over each sample and into four empty wells for the negative control. The plate was kept at room temperature on a 3D rotator plate for 24 h, before the samples were removed and the solution was sampled. The peroxide in each solution sample was measured using a Pierce Quantitative Peroxide Assay Kit. Samples were diluted 0, 5, or 200 times to ensure a read within the working range of the assay. The assay results were read using a Cytation 5 imaging reader (BioTek, VT).

##### Average Needle Length Assessment

0%(–) MNAs were prepared as described above and allowed to desiccate overnight. Individual rows of needles were cut apart using a razor blade, and mounted at 90 degrees on a stub using conductive tape. Samples were imaged with a Hitachi TM‐1000 Tabletop Microscope. The needles were measured using the scale bar from the micrographs, and the average was calculated among seven needles.

##### Degradation Study

Cell strainers were used to hold the material samples and facilitate easy removal from solution and weighing. To minimize the weight of the strainer and best capture changes in the material, the top plastic ring was removed. The dry weight of the strainers was measured and to determine the background mass of the strainer to consider during sampling, each one was fully submerged in PBS, removed, and dried in the manner that would be used throughout the experiment. These masses were recorded, and then the strainers were fully dried before 100 μL material samples were pipetted onto the bottom surface. Samples were crosslinked with 3 min of blue light exposure and then fully desiccated. Each strainer containing a sample was measured, and the dry mass was subtracted to find the mass of the dry sample. All samples were then placed in 6‐well plates containing 2 U mL^−1^ solution of collagenase. At each of the time points, the samples were removed, dried with a Kimwipe, and massed. After 48 h, the collagenase solution was refreshed after each sampling.

##### SEM

To prepare for SEM imaging, MNAs were desiccated overnight and then freeze‐dried (FreeZone 2.5 L − 50 °C Benchtop, Labconco) to ensure all moisture was removed. The samples were mounted on a stub using conductive tape, and sputter coating in gold for 60 s at 20 mA (Vacuum Desk V, Denton). Samples were imaged using a Hitachi TM‐1000 Tabletop Microscope.

##### Compression Testing

MNAs were fabricated and then desiccated overnight to eliminate any moisture. Samples (at least six per group) were placed on the bottom platen of a TA Electroforce 3220‐AT Series III mechanical tester, and the top platen was manually lowered until it had just contacted the tops of the needles. MNAs were compressed to 30% at rate of 0.1 mm s^−1^ with force to be measured as a function of displacement. The maximum compressive force created by each MNA was then identified.

##### MNA Swelling Tests

Three 0%(–) MNA samples were prepared and desiccated overnight. They were massed in their dry state, and then soaked in PBS for 24 h. At the timepoints shown, the MNAs were lifted out of the PBS, dabbed with a Kimwipe to remove excess liquid, and massed again. The values presented represent the percent change in mass from the initial time point to the measured time point.

##### Hydrogen Peroxide Release Assessment

30 μL material samples were prepared with the established CPO and catalase concentrations and placed in 500 μL of PBS and maintained at room temperature on a 3D rotator plate for the duration. Four replicates were used for each group. The full volume of PBS was sampled and refreshed at each time point. Samples were diluted 25 times to bring the concentration within the working range of a Pierce Quantitative Peroxide Assay Kit. The results of this colorimetric oxidation‐based assay were read on a Cytation 5 imaging reader (BioTek, VT).

##### Oxygen Release Evaluation

2 mL MNAs were fabricated and placed into bottles containing 20 mL of PBS, which were closed immediately. Three replicates were prepared for each of the eight groups. The bottle threads were wrapped with polytetrafluoroethylene sealing tape and the outsides of the bottles were sealed with Parafilm to minimize any gas exchange with the outside environment. The bottles were placed on a 3D platform rotator to maintain the homogeneity of the solution and remained sealed at room temperature for the 2 day release study. Touchless oxygen sensor dots and a four‐channel logger (PyroScience, Germany) were used to measure the concentration of oxygen at the selected time points. An ≈2 min reading was taken of each replicate in groups of four, and the first 110 time points were averaged to calculate the value of that sample.

##### In Vitro Cytocompatiblity Assessment

HDFs were cultured in 48 well plates and allowed to attach overnight before 30 μL crosslinked material samples were placed in the culture media. Four replicates were used per group. A PrestoBlue viability assay and Live/Dead imaging were performed according to manufacturer protocols 1 and 3 days after the introduction of samples. PrestoBlue results were read on the Cytation 5 imaging reader and Live/Dead images were captured using a Zeiss Axio Observer Inverted Microscope.

##### Cell Preparation for RNAseq

HDFs were seeded in 6‐well plates, with two wells paired to make up a single replicate worth of cells. Three total replicates were used for each group. The cells were incubated for one day before the plates in the hypoxia groups were moved to a 5% O_2_ incubator. One plate was maintained in a standard incubator as a positive control. After a day of hypoxia exposure, 100 μL material samples (0%(+) and 1%(+)) were prepared and exposed to the culture media using cell culture inserts. An untreated hypoxia group was maintained as a negative control. Cells were incubated with the samples for 2 days, and then lifted with Trypsin so they could be lysed.

##### Transcriptomics

The cells were lysed using buffer RLT, and the RNA was isolated with the RNeasy Mini Kit from Qiagen, as per the manufacturer's guidelines. The quality of the RNA was assessed using the Bioanalyzer 2100 from Agilent and only samples with a RIN value higher than 8 were used for the library preparation. The library preparation and RNA sequencing were performed by Novogene Inc. mRNA libraries prepared from the samples were sequenced on the Illmina platform. RNAseq reads were aligned to the human transctiptome (GRCh v38) using hisat2.^[^ ^33^ ^]^ The paired reads uniquely and concordantly aligning against a single gene were counted using feature Counts.^[^ ^34^ ^]^ The differential expression *p*‐values and fold changes were calculated using DESeq2^[^ ^35^ ^]^ on the R platform. Custom R scripts were used for downstream analysis to calculate gene ontology and KEGG gene‐set enrichment, *z*‐scores, and heatmaps.

##### Hypoxia Cell Rescue Studies

HDFs were split after their initial passage from thawing into hypoxic and normoxic lines. Cells in the hypoxic line were expanded for use, plated, attached, and stored in a 5% O_2_ incubator and always treated with culture media that had been incubated in low oxygen for at least 24 h. Cells in the normoxic line were kept in a standard 20% O_2_ incubator in the lead‐up to and for the duration of the experiment and were always treated with standard media stored in sealed tubes in the refrigerator. After expansion, the cells were cultured in 48‐well plates first thing in the morning to allow the cells to attach while experimental samples were fabricated. 30 μL material samples (four per group) were prepared and added to the culture media in the afternoon of the same day. The plates were returned to their respective incubators overnight and a PrestoBlue viability assay was performed one and two days after the samples were applied according to manufacturer protocol.

Human umbilical vein endothelial cells were plated into 48‐well plates and allowed to attach overnight before half were transferred into the hypoxic incubator for an additional day. On the second day after plating, 30 μL material samples were fabricated and added to the media. Plates were returned to the normoxic and hypoxic incubators and a PrestoBlue viability assay was run three days after the addition of the material samples.

##### Animal Study

All animal procedures were approved by the Institutional Animal Care and Use Committee of University of Connecticut Health Center and were performed in compliance with the National Institutes of Health guidelines. 8–10 week old female C57BL/6 mice were obtained from Jackson Laboratories and were acclimatized for 1 week before surgery. The animals were given ad libitum access to food and water. The weight of the animals was monitored throughout the study to ensure their general health.

Depilation was performed 1 day before the surgeries. Under general anesthesia with isoflurane, the skin was shaved using a clipper followed by application of depilatory cream to remove hair. Subsequently, the skin was cleaned with gauze. The following day (day 0), the animals were anesthetized, the skin was cleaned with an ethanol wipe, and a full thickness circular wound was created on the dorsal skin of each mouse using a 10 mm biopsy punch. The cut passes through the panniculus carnosus muscle to prevent wound closure through contraction. After wound creation, the treatments were applied and the wound was covered with a transparent and semiocclusive dressing (Tegaderm, 3M, St. Paul, MN). Four different treatments were applied (*n* = 5): 1) no treatment (negative control); 2) plain GelMA MNAs (0%(–)); 3) GelMA MNAs containing catalase (0%(+)); and 4) GelMA MNAs supplemented with 1% w/v catalase and 1% w/v CPO (1%(+)). The animals were then placed under a warming light until they were fully recovered and then recaged.

The wound closure was monitored on days 3, 7, and 10 postsurgery by removing the Tegaderm, imaging the wounds and placing new Tegaderm to cover the area. On day 10, the animals were euthanized, and the wounds with surrounding tissue were harvested for downstream histology and immunofluorescence staining.

##### Wound Closure Analysis

Images captured during the animal study were analyzed using ImageJ (U. S. National Institutes of Health, Bethesda, Maryland, USA) to quantify the area. The ruler in each image was used to fix the number of pixels that represented 1 cm, and then the wounded area in cm^2^ was measured using the polygon tool. The percent wound closure was determined by calculating the percent change from the initial wound area.

##### Histology

Tissue was fixed in 4% paraformaldehyde overnight before being embedded in OCT and sectioned. H&E and Masson Trichrome staining was performed according to the manufacturer's protocols. For CD31 immunofluorescent staining, slides were soaked in 1X PBS for 10 min and then placed in an antigen retrieval citrate buffer at 60 °C overnight. The next day, they were blocked for 1 h using 5% normal goat serum and then exposed to the primary antibody (LS‐B5577‐50, Lifespan Biosciences), diluted 1:100, for 2 h at room temperature in 5% normal goat serum working solution. In a light proof container, the secondary antibody (A32731, ThermoFisher Scientific) was then applied for 3 h at room temperature at a dilution of 1:1000. DAPI, at a concentration of 1:1000, was applied in the mounting medium as the sections were cover‐slipped. Three rinses of 5 min each in 1X PBS were performed between each step.

##### CD31 Staining Quantification

CD31 image analysis was done using FIJI (U. S. National Institutes of Health, Bethesda, Maryland, USA). The number of bright pixels in the green channel above a certain threshold was counted in and around the wound area as a measure of CD31 expression and compared to uninjured adjacent tissue on the same section. Two rectangular regions of interest (ROI) of the same size were defined for each image: one to cover the wound area and one to cover healthy tissue. A threshold for the green channel was set to exclude the background noise from the measurement. The same threshold was used for all images. The number of bright pixels in the ROIs which were above the set threshold was counted and reported.

##### Statistical Analysis

Statistical analyses were performed utilizing GraphPad Prism 6.0 software (Graph Pad Software Inc., La Jolla, CA). Comparisons of the different groups were performed using a one‐way ANOVA with Tukey's multiple comparison test or a two‐way ANOVA with Sidak's multiple comparison test when there were two input variables (when comparing three or more groups) or *T*‐test (when comparing two groups), and (adjusted) *P*‐values smaller than 0.05 were considered statistically significant. All of the experiments were performed at least in triplicates and data were presented as mean ± standard deviation. *, **, ***, and **** represent *P* < 0.05, *P* < 0.01, *P* < 0.001, and *P* < 0.0001, respectively.

## Conflict of Interest

J.Q., A.T., and M.S. are co‐founders of InPrint Bio.

## Author Contributions

**Lindsay Barnum**: Conceptualization (supporting); Formal analysis (lead); Investigation (lead); Methodology (lead); Writing—original draft (lead); Writing—review & editing (lead). **Mohamadmahdi Samandari**: Conceptualization (supporting); Investigation (supporting); Supervision (supporting); Writing—original draft (supporting); Writing—review & editing (supporting). **Yasir Suhail**: Data curation (supporting); Formal analysis (supporting); Writing—original draft (supporting); Writing—review & editing (supporting). **Steven Toro**: Formal analysis (supporting); Methodology (supporting); Visualization (supporting); Writing—review & editing (supporting). **Ashkan Novin**: Data curation (supporting); Investigation (supporting); Methodology (supporting); Writing—review & editing (supporting). **Pejman Ghelich**: Formal analysis (supporting); Investigation (supporting); Methodology (supporting). **Jacob Quint**: Conceptualization (supporting); Formal analysis (supporting); Writing—review & editing (supporting). **Farnooosh Saeedinejad**: Formal analysis (supporting); Investigation (supporting); Writing—review & editing (supporting). **Manu Komma**: Investigation (supporting); Visualization (supporting). **Kshitiz**: Formal analysis (supporting); Supervision (supporting); Visualization (supporting); Writing—review & editing (supporting). **Ali Tamayol**: Conceptualization (lead); Funding acquisition (lead); Methodology (supporting); Writing—original draft (supporting); Writing—review & editing (supporting).

## Supporting information

Supplementary Material

## Data Availability

The data that support the findings of this study are available from the corresponding author upon reasonable request.

## References

[1] R. Choudhury , Int. J. Gen. Med. 2018, 11, 431. 30538529 10.2147/IJGM.S172460 PMC6251354

[2] H. M. Kimmel , A. Grant , J. Ditata , Wounds 2016, 28, 264. 27560469

[3] D. M. Castilla , Z.‐J. Liu , O. C. Velazquez , Adv. Wound Care 2012, 1, 225. 10.1089/wound.2011.0319 PMC3625368 24527310

[4] S. Singh , A. Young , C.‐E. McNaught , Surgery 2017, 35, 473.

[5] J. A. Thackham , D. L. S. McElwain , R. J. Long , Wound Repair Regener. 2008, 16, 321. 10.1111/j.1524-475X.2008.00372.x 18471250

[6] G. M. Gordillo , C. K. Sen , Int. J. Lower Extremity Wounds 2009, 8, 105. 10.1177/1534734609335149 PMC3814219 19443899

[7] P. Kranke , M. H. Bennett , M. Martyn‐St James , A. Schnabel , S. E. Debus , S. Weibel , Cochrane Database Syst. Rev. 2015, 2015, CD004123. 26106870 10.1002/14651858.CD004123.pub4 PMC7055586

[8] B. Hajhosseini , B. A. Kuehlmann , C. A. Bonham , K. J. Kamperman , G. C. Gurtner , Plast. Reconst. Surg. – Global Open 2020, 8, e3136. 10.1097/GOX.0000000000003136 PMC7544320 33133975

[9] T. E. Serena , N. M. Bullock , W. Cole , J. Lantis , L. Li , S. Moore , K. Patel , M. Sabo , N. Wahab , P. Price , J. Wound Care 2021, 30, S7. 33979229 10.12968/jowc.2021.30.Sup5.S7

[10] J. Yu , S. Lu , A.‐M. McLaren , J. A. Perry , K. M. Cross , Wound Repair Regener. 2016, 24, 1066. 10.1111/wrr.12490 27733020

[11] a) D. P. Ntentakis , A. M. Ntentaki , E. Delavogia , L. Kalomoiris , D. Venieri , N. Arkadopoulos , N. Kalogerakis , Wound Repair Regener. 2021, 29, 1062; 10.1111/wrr.12972 34655455

[12] H. Wang , Y. Zhao , T. Li , Z. Chen , Y. Wang , C. Qin , Chem. Eng. J. 2016, 303, 450.

[13] L. Barnum , M. Samandari , T. A. Schmidt , A. Tamayol , Expert Opin. Drug Delivery 2020, 17, 1767. 10.1080/17425247.2020.1819787 PMC7722049 32882162

[14] E. Lazarus , L. Barnum , S. Ramesh , J. Quint , M. Samandari , S. Laflamme , T. W. Secord , T. Schmidt , A. Tamayol , I. V. Rivero , Appl. Phys. Rev. 2024, 11, 021301.

[15] a) M. Samandari , J. Quint , A. Rodríguez‐delaRosa , I. Sinha , O. Pourquié , A. Tamayol , Adv. Mater. 2021, 34, 2105883; 10.1002/adma.202105883 PMC8957559 34773667

[16] L. Atkin , Br. J. Community Nurs. 2019, 24, S26. 10.12968/bjcn.2019.24.Sup9.S26 31479336

[17] Y. Deng , C. Yang , Y. Zhu , W. Liu , H. Li , L. Wang , W. Chen , Z. Wang , L. Wang , Nano Lett. 2022, 22, 2702. 35324204 10.1021/acs.nanolett.1c04573

[18] Z. Yao , T. Xue , H. Xiong , C. Cai , X. Liu , F. Wu , S. Liu , C. Fan , Mater. Sci. Eng., C 2021, 119, 111446. 10.1016/j.msec.2020.111446 33321586

[19] J. Gan , X. Zhang , W. Ma , Y. Zhao , L. Sun , Nano Today 2022, 47, 101630.

[20] D. Ghanbariamin , M. Samandari , P. Ghelich , S. Shahbazmohamadi , T. A. Schmidt , Y. Chen , A. Tamayol , Small 2023, 19, 2207131. 10.1002/smll.202207131 PMC13382645 37026428

[21] W. Ma , X. Zhang , Y. Liu , L. Fan , J. Gan , W. Liu , Y. Zhao , L. Sun , Adv. Sci. 2022, 9, 2103317. 10.1002/advs.202103317 PMC9069192 35266637

[22] a) X. Zhang , G. Chen , Y. Liu , L. Sun , L. Sun , Y. Zhao , ACS Nano 2020, 14, 5901; 32315159 10.1021/acsnano.0c01059

[23] H. Sepasi Tehrani , A. A. Moosavi‐Movahedi , Prog. Biophys. Mol. Biol. 2018, 140, 5. 29530789 10.1016/j.pbiomolbio.2018.03.001

[24] a) F. Dadkhah Tehrani , I. Shabani , A. Shabani , Carbohydr. Polym. 2022, 281, 119020; 35074102 10.1016/j.carbpol.2021.119020

[25] a) K. Yue , G. Trujillo‐de Santiago , M. M. Alvarez , A. Tamayol , N. Annabi , A. Khademhosseini , Biomaterials 2015, 73, 254; 26414409 10.1016/j.biomaterials.2015.08.045 PMC4610009

[26] a) K.‐T. Chang , Y.‐K. Shen , F.‐Y. Fan , Y. Lin , S.‐C. Kang , J. Manuf. Processes 2020, 54, 274;

[27] a) S. Schreml , R. M. Szeimies , L. Prantl , S. Karrer , M. Landthaler , P. Babilas , Br. J. Dermatol. 2010, 163, 257; 20394633 10.1111/j.1365-2133.2010.09804.x

[28] Y. Fujiki , M. C. Bassik , Trends Cell Biol. 2021, 31, 148. 33422360 10.1016/j.tcb.2020.12.006

[29] H. M. Abdel‐Mageed , A. E. Abd El Aziz , B. M. Abdel Raouf , S. A. Mohamed , D. Nada , 3 Biotech 2022, 12, 73. 10.1007/s13205-022-03131-4 PMC8859020 35211369

[30] a) M. Hu , K. Korschelt , P. Daniel , K. Landfester , W. Tremel , M. B. Bannwarth , ACS Appl. Mater. Interfaces 2017, 9, 38024; 29019391 10.1021/acsami.7b12212

[31] A. Siddiqui , R. D. Galiano , D. Connors , E. Gruskin , L. Wu , T. A. Mustoe , Wound Repair Regener. 1996, 4, 211. 10.1046/j.1524-475X.1996.40207.x 17177815

[32] a) O. Burgy , M. Königshoff , Matrix Biol. 2018, 68–69, 67; 10.1016/j.matbio.2018.03.017 29572156

[33] D. Kim , J. M. Paggi , C. Park , C. Bennett , S. L. Salzberg , Nat. Biotechnol. 2019, 37, 907. 31375807 10.1038/s41587-019-0201-4 PMC7605509

[34] Y. Liao , G. K. Smyth , W. Shi , Bioinformatics 2013, 30, 923. 24227677 10.1093/bioinformatics/btt656

[35] M. I. Love , W. Huber , S. Anders , Genome Biol. 2014, 15, 550. 25516281 10.1186/s13059-014-0550-8 PMC4302049

[36] P. Ghelich , M. Samandari , A. Hassani Najafabadi , A. Tanguay , J. Quint , N. Menon , D. Ghanbariamin , F. Saeedinejad , F. Alipanah , R. Chidambaram , R. Krawetz , K. Nuutila , S. Toro , L. Barnum , G. D. Jay , T. A. Schmidt , A. Tamayol , Adv. Healthcare Mater. 2024, 13, 2302836. 10.1002/adhm.202302836 38299437

