# Supplementary material for: Biodegradable Oxygen‐Generating Microneedle Patches for Regenerative Medicine Applications
Source: Adv Nanobiomed Res. 2024 Nov 27;5(1):2400093. doi: 10.1002/anbr.202400093 (PMC12077069; doi:10.1002/anbr.202400093)
Supplement: Supplementary file 1 — Supplementary Material [file ANBR-5-2400093-s001.pdf]

Supplementary Materials

Biodegradable oxygen-generating microneedle patches for regenerative medicine applications

Lindsay Barnum[1,2], Mohamadmahdi Samandari[1], Yasir Suhail[1,2], Steven Toro[1], Ashkan Novin[1] Pejman Ghelich[1,2], Jacob Quint[1,2], Farnooosh Saeedinejad[1,2], Kshitiz[1,2], Ali Tamayol[1,2,*]

[1]Department of Biomedical Engineering, University of Connecticut Health Center, Farmington, CT 06030, USA

[2]Department of Biomedical Engineering, University of Connecticut, Storrs, CT 06269, USA

[*] Corresponding author: A. Tamayol (atamayol@uchc.edu)

Supplementary Figures:

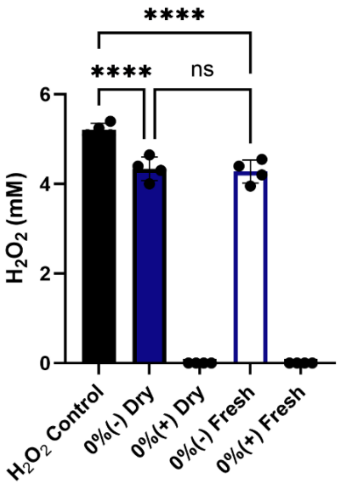

Figure S1: Results of peroxide assay after 5 mM stock solution was treated with dry and fresh 0%(-) and 0%(+) samples.

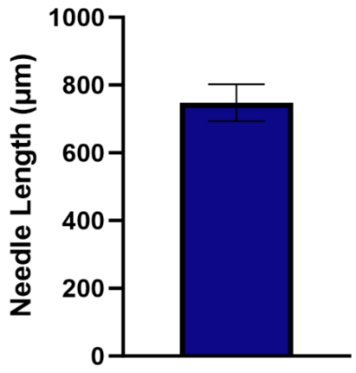

Figure S2: Average length of microneedles after shrinking through desiccation process.

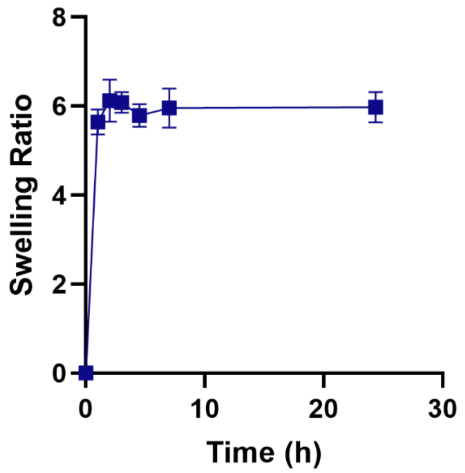

Figure S3: Swelling ratio of plain GelMA MNA samples over 24 hours.

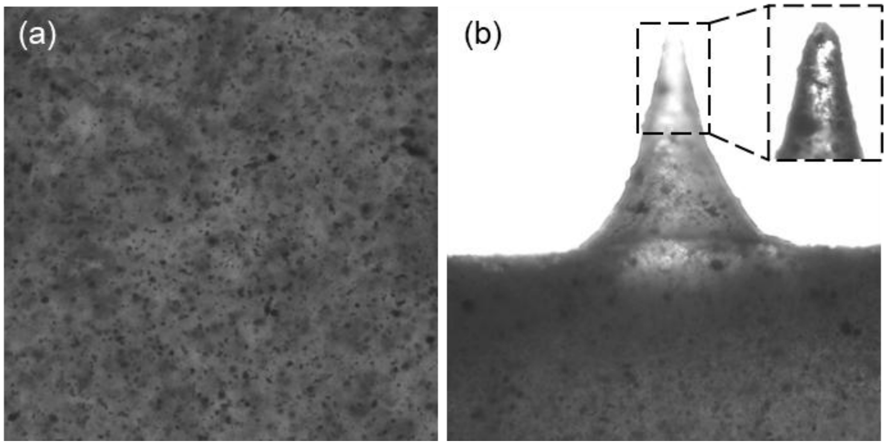

Figure S4: Distribution of CPO particles in (a) a top view of the backing and (b) a side view of the backing and a needle. Inset image shows zoomed in view of needle tip in focus.

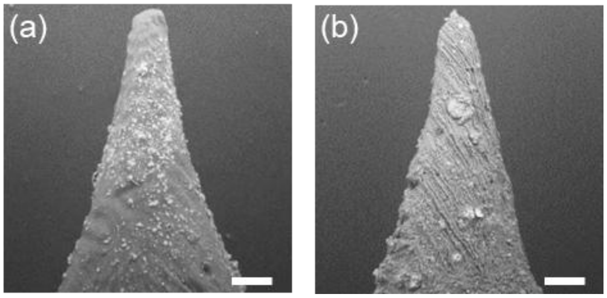

Figure S5: Scanning electron microscopy (SEM) images of a single (a) 0%(-) and (b) 2%(+) microneedle, respectively, showing similar structure.

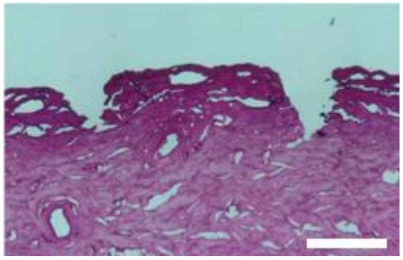

Figure S6: H&E-stained tissue section showing needle penetration through the dermis. Scale bar represents 500 µm

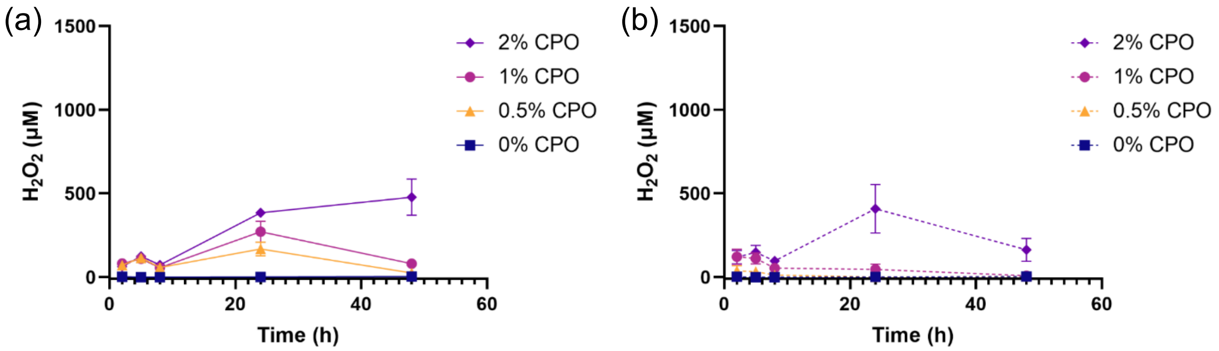

Figure S7: H_2O_2 present at each time point over 48 hours (a) without catalase and (b) with catalase.

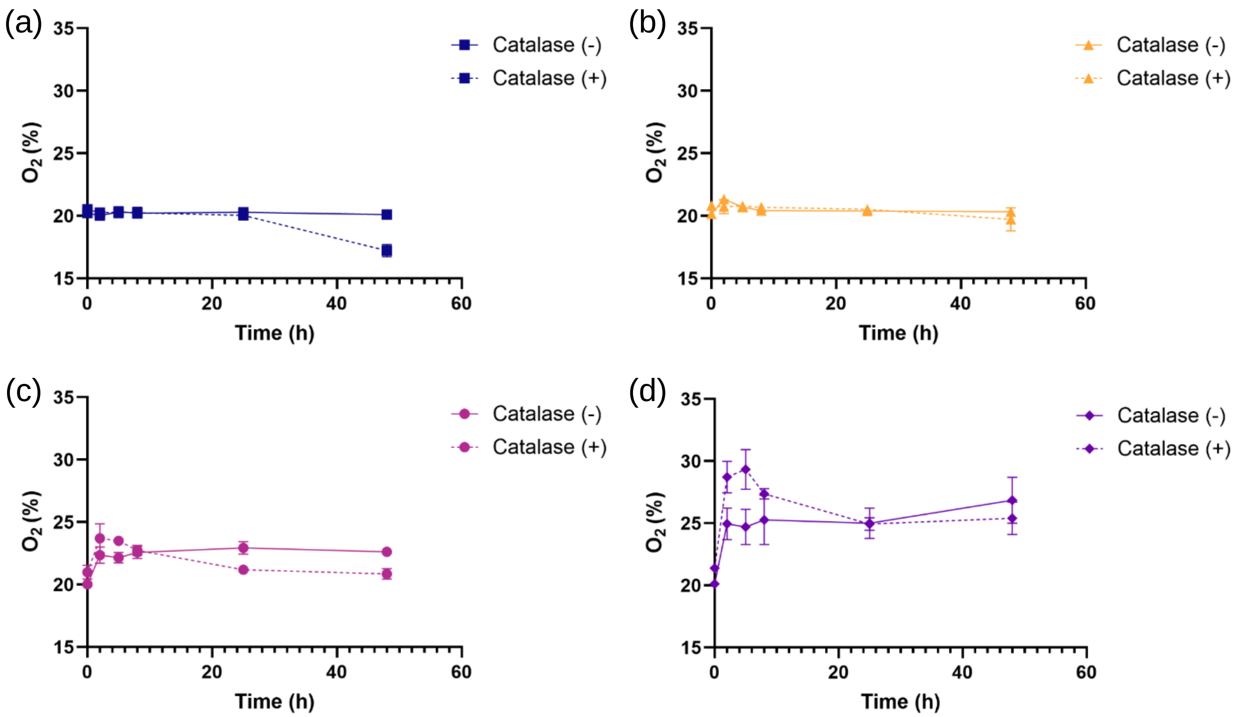

Figure S8: Alternate presentation of Figure 2i and j, showing oxygen release from samples with and without catalase for the four concentrations of CPO studied: (a) 0%, (b) 0.5%, (c) 1%, and (d) 2%.

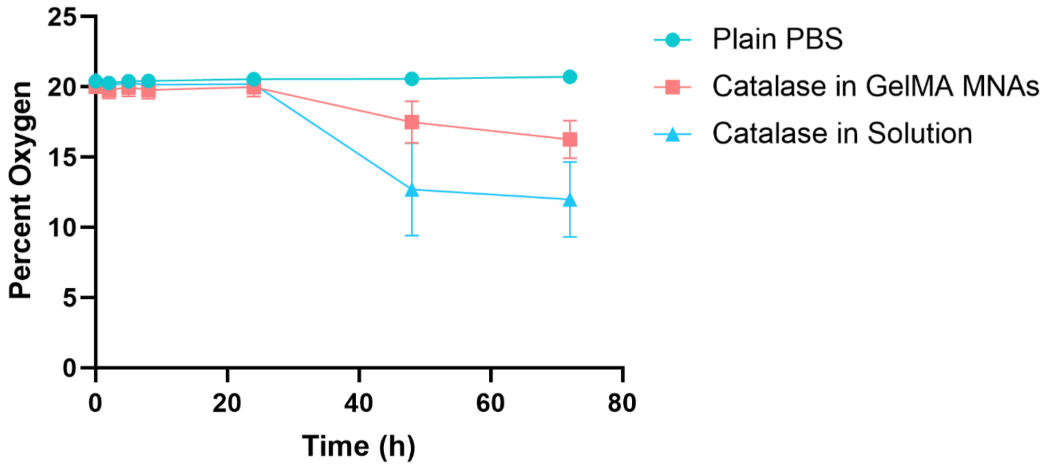

Figure S9: Drop in oxygen concentration when catalase is introduced to the solution.

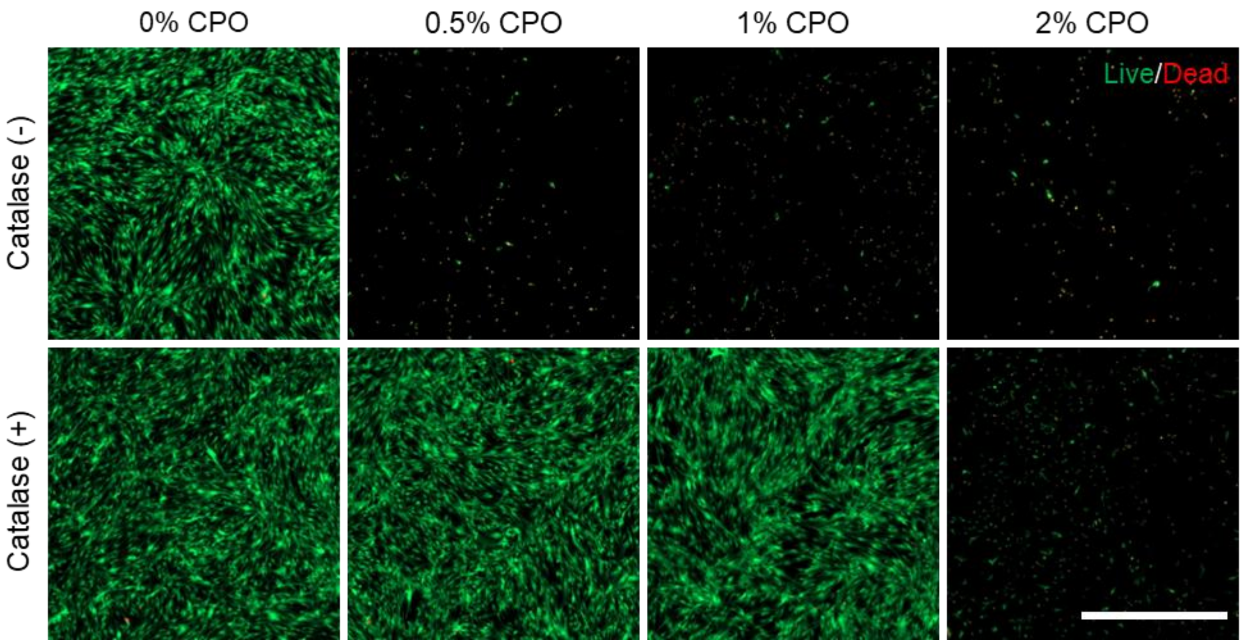

Figure S10: Representative Day 3 Live/Dead imaging of HDFs exposed on material samples. Scale bar represents 500µm.

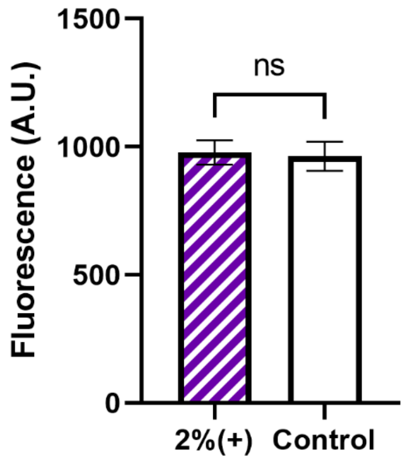

Figure S11: Background testing of most complex treatment sample compared to PrestoBlue control.

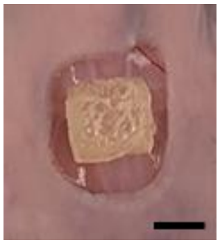

Figure S12: Representative image showing 1%(+) MNA placed on the back of wounded mouse. Scale bar represents 500 µm.

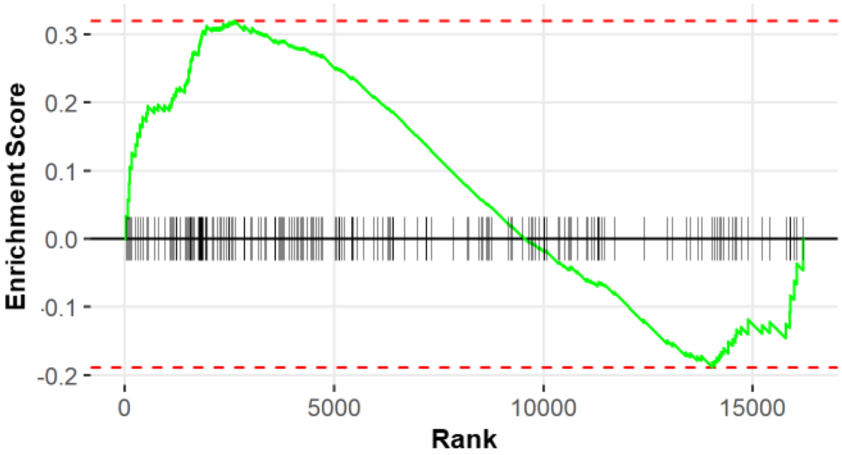

Figure S13: Gene set enrichment analysis showing upregulation in the KEGG pathway for the regulation of actin cytoskeleton.

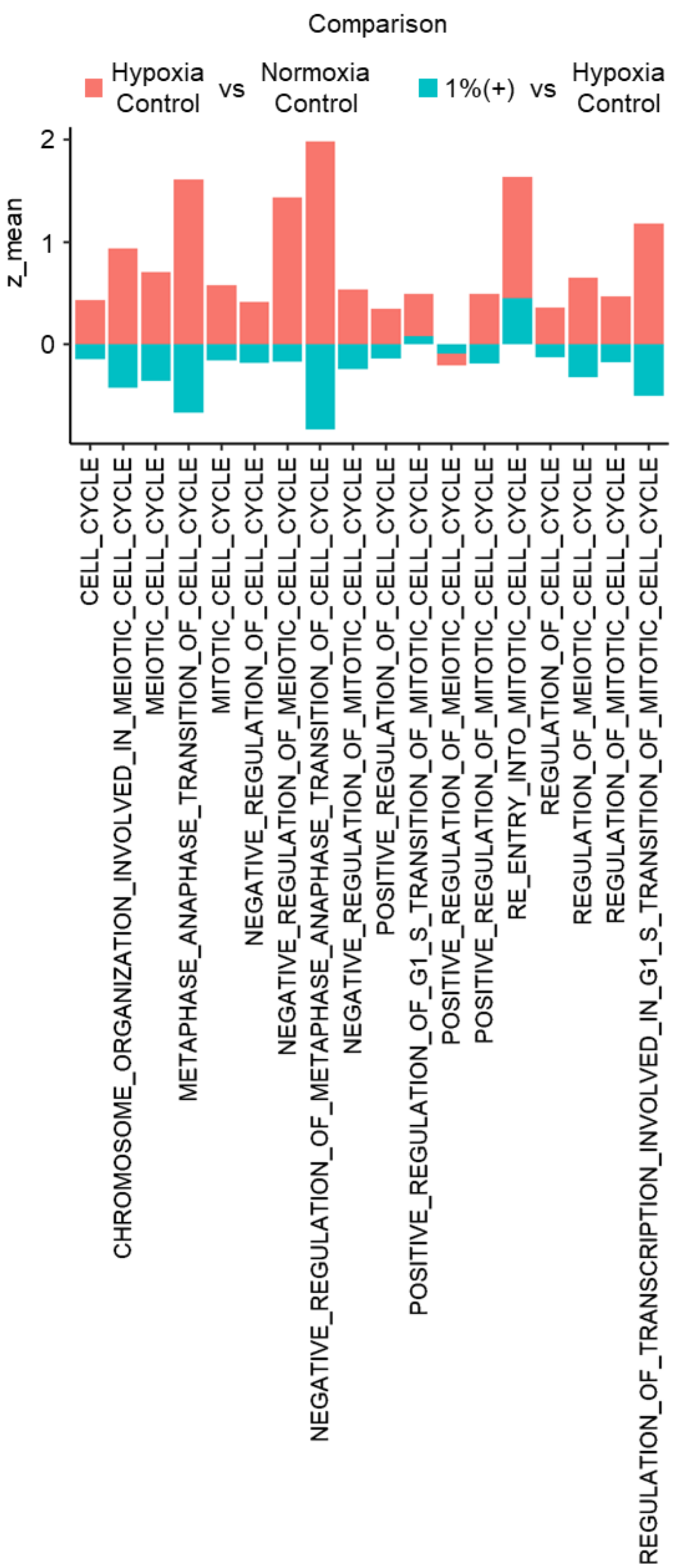

Comparison
Hypoxia Control vs Normoxia Control
1%(+) vs Hypoxia Control
z_mean
2
1
0
CELL_CYCLE
CHROMOSOME_ORGANIZATION_INVOLVED_IN_MEIOTIC_CELL_CYCLE
MEIOTIC_CELL_CYCLE
METAPHASE_ANAPHASE_TRANSITION_OF_CELL_CYCLE
MITOTIC_CELL_CYCLE
NEGATIVE_REGULATION_OF_CELL_CYCLE
NEGATIVE_REGULATION_OF_MEIOTIC_CELL_CYCLE
NEGATIVE_REGULATION_OF_METAPHASE_ANAPHASE_TRANSITION_OF_CELL_CYCLE
NEGATIVE_REGULATION_OF_MITOTIC_CELL_CYCLE
POSITIVE_REGULATION_OF_CELL_CYCLE
POSITIVE_REGULATION_OF_G1_S_TRANSITION_OF_MITOTIC_CELL_CYCLE
POSITIVE_REGULATION_OF_MEIOTIC_CELL_CYCLE
POSITIVE_REGULATION_OF_MITOTIC_CELL_CYCLE
RE_ENTRY_INTO_MITOTIC_CELL_CYCLE
REGULATION_OF_CELL_CYCLE
REGULATION_OF_MEIOTIC_CELL_CYCLE
REGULATION_OF_MITOTIC_CELL_CYCLE
REGULATION_OF_TRANSCRIPTION_INVOLVED_IN_G1_S_TRANSITION_OF_MITOTIC_CELL_CYCLE

Figure S14: Cell proliferation related gene ontology sets, and the mean regulation comparing hypoxia control to normoxia control and the 1%(+) treated group in hypoxia to control show a CPO induced reversal in the changes accompanied in hypoxia.

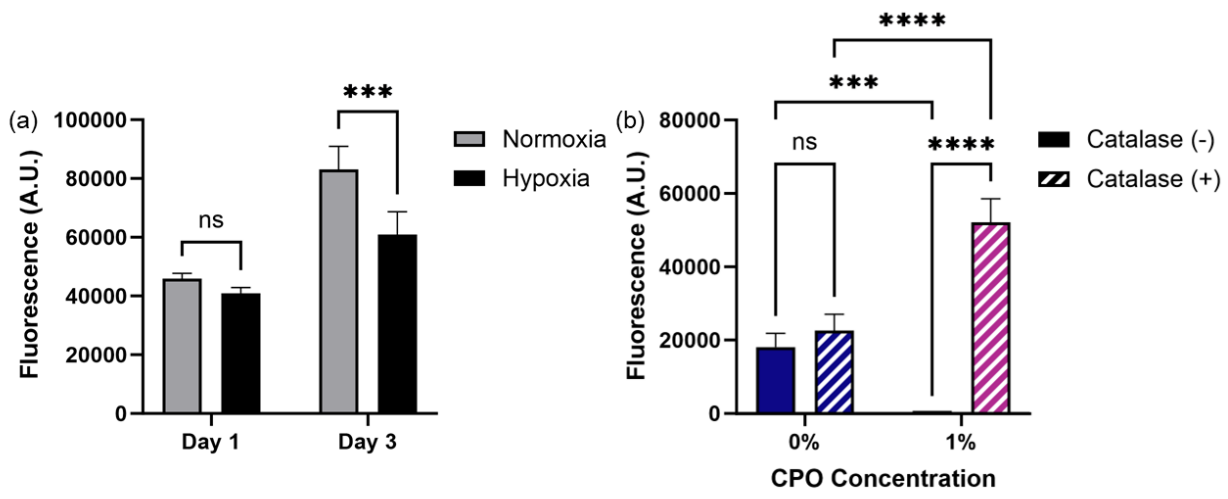

Figure S15: Human umbilical vein endothelial cell response to hypoxia and treatment. (a) PrestoBlue signal after one and three days of culture in 20% and 5% O_2 incubator shows lower viability in hypoxia. (b) PrestoBlue results after three days of culture in 5% O_2 incubator with 0% and 1% CPO material samples. Treatment with 1%(+) sample significantly improves viability over 0%(+) group.

Table S1: Summary of P-values for hydrogen peroxide release.

	(-) Catalase					(+) Catalase				
	2 hours	5 hours	8 hours	24 hours	48 hours	2 hours	5 hours	8 hours	24 hours	48 hours
0% CPO vs. 0.5 % CPO	****	**	***	***	**	ns	ns	ns	ns	ns
0% CPO vs. 1% CPO	***	***	****	***	**	*	*	*	*	*
0% CPO vs. 2% CPO	****	****	****	****	**	*	*	*	*	*
0.5% CPO vs. 1 % CPO	ns	ns	ns	ns	*	ns	ns	*	*	*
0.5% CPO vs. 2 % CPO	ns	ns	ns	***	**	ns	*	*	*	*
1% CPO vs. 2% CPO	**	ns	ns	*	**	ns	ns	ns	ns	ns

Table S2: Summary of P-values for oxygen release.

	(-) Catalase						(+) Catalase					
	0 h	2 h	5 h	8 h	24 h	48 h	0 h	2 h	5 h	8 h	24 h	48 h
0% CPO vs. 0.5% CPO	ns	*	ns	ns	ns	ns	ns	ns	ns	ns	ns	ns

0% CPO vs. 1% CPO	ns	*	*	*	**	**	ns	ns	*	**	ns	**
0% CPO vs. 2% CPO	ns	*	ns	ns	*	ns	*	*	*	***	**	*
0.5% CPO vs. 1% CPO	ns	ns	*	*	*	*	ns	ns	**	*	ns	ns
0.5% CPO vs. 2% CPO	ns	ns	ns	ns	ns	ns	ns	**	*	***	**	*
1% CPO vs. 2% CPO	ns	ns	ns	ns	ns	ns	ns	*	ns	***	**	ns
